# Drug eruption induced by the intake of comprehensive cold medicine: A case report

**DOI:** 10.1097/MD.0000000000044357

**Published:** 2025-09-05

**Authors:** Yimin Huang, Yingying Tang, Na Huang, Mengya Shen, Minyan Qian, Li Ni

**Affiliations:** a Department of Clinical Pharmacy, Jiaxing Maternity and Child Health Care Hospital, Affiliated Women and Children Hospital, Jiaxing University, Jiaxing, Zhejiang, China; b Department of Pediatrics, Jiaxing Maternity and Child Health Care Hospital, Affiliated Women and Children Hospital, Jiaxing University, Jiaxing, Zhejiang, China.

**Keywords:** adverse effect, drug-related, Taisho Comprehensive Cold Medicine

## Abstract

**Rationale:**

Taisho Comprehensive Cold Medicine is a Japanese drug known for its effective action against the common cold. It was widely used by the Chinese individuals. Meanwhile, the cold medicine is associated with several adverse effects. Although some drug eruptions induced by paracetamol have been reported, those induced by comprehensive cold medicine have not been studied.

**Patient concerns:**

A 7-year-old child presenting with an upper respiratory tract infection developed drug eruptions with symptoms of significant pruritus and skin rash, after 2 days of therapy with Taisho Comprehensive Cold Medicine.

**Diagnoses:**

The clinical symptoms and the laboratory examination confirmed drug eruption, accompanied by mild renal impairment. And Taisho Comprehensive Cold Medicine-induced drug eruption and renal impairment was considered. The Naranjo Adverse Drug Reaction Probability Scale score was 7 points, which is categorized as “probable.”

**Interventions:**

The doctor ceased the treatment of cold medicine and monitored the patient daily.

**Outcomes:**

The drug eruption disappeared 5 days after drug withdrawal.

**Lessons:**

Although Taisho Comprehensive Cold Medicine has been approved for its use in children over the age of 1 year, it is necessary to clarify whether children under the age of 18 have a history of allergies when prescribing or administering this medicine. And medication should be taken under the supervision of a doctor. Once adverse reactions occur, stop taking the medicine immediately. This is the 1st known case of Taisho Comprehensive Cold Medicine-induced drug eruption. The awareness of rare adverse reaction helps ensure the clinical safety of Taisho Comprehensive Cold Medicine treatment in Chinese children.

## 1. Introduction

Adverse drug reaction (ADR) refers to any harmful or unpleasant reaction following the administration of 1 or more drugs at normal dose(s). This reaction in the body may result from the drug itself, or be an interaction between several drugs, with no purpose to prevent, diagnose, or treat a disease, or even to recover the patient’s physiological/physical functions after suffering from a disease/condition.^[1]^ If the ADR manifests mainly as an acute inflammation on the skin and/or mucosa, it is called a drug eruption or dermatitis medicamentosa.^[2]^ Antiepileptic, antibiotic, nonsteroidal, and anti-inflammatory drugs can cause 1% to 8% of drug eruptions; others such as acetaminophen, antihistamines, and steroid hormones rarely induce such reactions, according to post-marketing analyses.^[3,4]^ Most drug eruptions are benign, but approximately 2% lead to serious and life threatening episodes such as acute generalized exanthematous pustulosis, toxic epidermal necrolysis, and Stevens–Johnson syndrome.^[5]^

Most drugs exert their pharmacological effects on the body through the production of metabolites, which is derived from a series of metabolic reactions. The skin contains many cells such as neutrophils, monocytes, and keratinocytes, which participates in drug metabolism. Furthermore, the skin is an immune-active organ containing Langerhans and dendritic cells, which presents drug antigenic determinants to immune effector cells. Therefore, the skin is the most commonly affected organ during drug reactions (ADRs).^[6]^

It is unclear whether the mechanism of drug eruption is mediated by the immune system or nonallergic reactions. However, according to a previous study, intraepidermal CD8^+^ T cells may contribute to the development of localized epidermal lesions.^[7]^

There are only a few published reports about drug eruptions induced by paracetamol but none by cold medicine ingredients. Herein, we report the case of drug eruptions developing on the extremities of a child after receiving Comprehensive Cold Medicine manufactured by Taisho Pharmaceutical Co., Ltd., (Tokyo, Japan).

## 2. Case description

**Figure 1.**
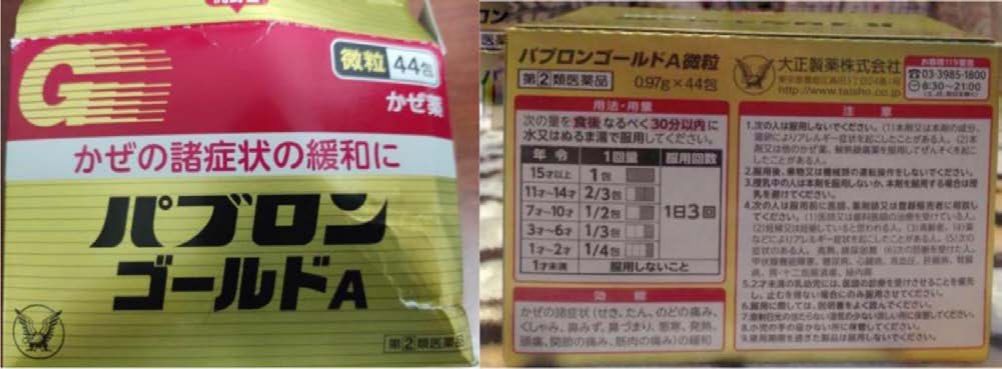
Comprehensive cold medicine manufactured by Taisho Pharmaceutical Co., Ltd (showed by patient’s parents).

A 7-year-old Chinese male child presented to the dermatologist with a 2-day history of rash and pruritus. Prior to the visit, the child had taken Taisho Comprehensive Cold Medicine, which was purchased from Japan by his parents, for an upper respiratory tract infection. The medication was administered twice daily at half a packet per dose. However, according to the drug’s instructions, the recommended dosage for children of this age is 3 times daily, with half a packet per dose (Fig. 1).

**Figure 2.**
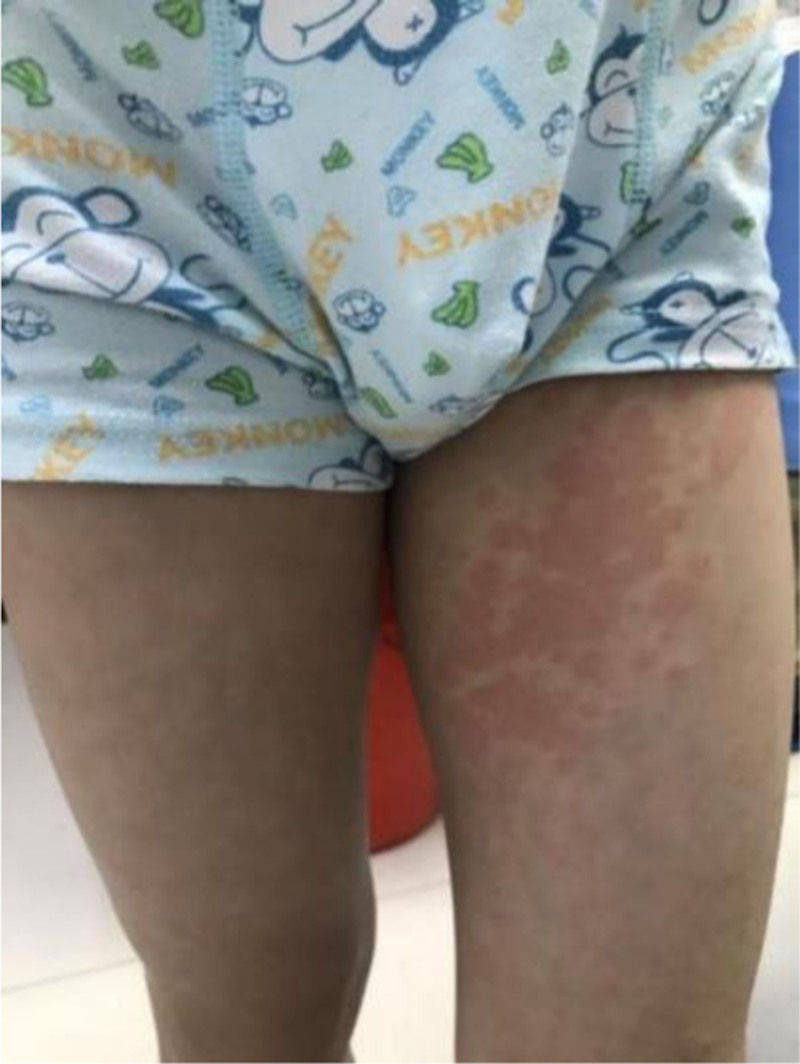
Clinical manifestation showing erythematous plaques on the patient’s front side of left thigh.

At the time of presentation, the child’s body temperature was 36.9°C. Physical examination revealed large round erythematous patches and papules on the trunk and limbs (Fig. 2). The dermatologist ordered urinalysis, biochemical analysis, complete blood count, hypersensitivity testing, and serum amyloid A protein testing. Urinalysis showed proteinuria (±) (normal result should be negative) and abnormal urobilinogen levels (±) (normal result should be negative), suggesting mild renal injury. Biochemical analysis demonstrated indirect bilirubin at 2.3 μmol/L (reference range: 3.4–14.2 μmol/L), serum creatinine at 43 μmol/L (reference range: 62–106 μmol/L), uric acid at 197 μmol/L (reference range: 210–420 μmol/L), serum glucose at 6.12 mmol/L (reference range: 3.89–6.11 mmol/L), and triglycerides at 1.87 mmol/L (reference range: 0.50–1.70 mmol/L). Complete blood count, hypersensitivity tests, and serum amyloid A protein levels were within normal ranges. Given the young age of the patient, skin biopsy was not performed. The patient had no previous history of drug allergy, skin diseases, or other allergic diseases, and there was no other relevant medication history. Based on the clinical manifestations and evidence of renal impairment, the dermatologist attributed the rash to an adverse effect of the Taisho Comprehensive Cold Medicine, as no other confounding factors could account for the cutaneous findings. The child was diagnosed with allergic dermatitis and admitted for further management.

The symptoms that originally presented upon admission to the hospital disappeared 5 days after drug withdrawal. The patient’s parents were advised to avoid the use of foreign cold medicines due to the possibility of unknown adverse reactions occurring. During the telephone follow-up, the patient reported no recurrence of symptoms since the discontinuation of the cold medicine. According to the Naranjo Adverse Drug Reaction Probability Scale, the score was 7 points, which falls into the category of “probable” (Table 1). This suggests that it is highly likely the Taisho Comprehensive Cold Medicine caused the symptoms observed in the patient. And this ADR was therefore reported to the Chinese National Adverse Reaction Monitoring Center in April 2020.

**Table 1:** Naranjo adverse drug reaction probability scale.

Factors	Yes	No	Do not know	Score
1. Are there previous conclusive reports on this reaction?	+1	0	0	+1
2. Did the adverse event appear after the suspected drug was administered?	+2	−1	0	+2
3. Did the adverse reaction improve when the drug was discontinued or a specific antagonist was administered?	+1	0	0	+1
4. Did the adverse reaction reappear when the drug was readministered?	+2	−1	0	0
5. Are there alternative causes (other than the drug) that could on their own have caused the reaction?	−1	+2	0	+2
6. Did the reaction reappear when a placebo was given?	−1	+1	0	0
7. Was the drug detected in the blood (or other fluids) in concentrations known to be toxic?	+1	0	0	0
8. Was the reaction more severe when the dose was increased, or less severe when the dose was decreased?	+1	0	0	0
9. Did the patient have a similar reaction to the same or similar drugs in any previous exposure?	+1	0	0	0
10. Was the adverse event confirmed by any objective evidence?	+1	0	0	+1
Total				7

9 points >: definite, 5 to 8 points: probable, 2 to 4 points: possible, 2 points <: doubtful.

## 3. Discussion

With the development of the economy in China, foreign medicines, such as the Taisho Comprehensive Cold Medicine, are becoming increasingly available to the Chinese population. Unfortunately, this population may not understand the instructions for the ingredients, use, and administration of foreign medications due to the lack of patient package inserts in Chinese. Hence, these are frequently misused, resulting in a subsequent increase in the number of ADRs.

Taisho Comprehensive Cold Medicine is a Japanese drug known for its effective action against the common cold. It is composed of 9 ingredients, and 1 packet includes acetaminophen (300 mg), dihydrocodeine phosphate (8 mg), ephedrine hydrochloride (20 mg), guaifenesin (41.67 mg), lysozyme hydrochloride (20 mg), carbinoxamine maleate (2.5 mg), caffeine anhydrous (25 mg), Vitamin B1 (8 mg), and riboflavin (4 mg). However, several of these ingredients should be avoided by children. An early clinical investigation found that children with acute codeine intoxication would develop 1 or more symptoms such as rashes, swelling, itching, somnolence, vomiting, and ataxia.^[8]^ Furthermore, its effectiveness and safety have not been demonstrated by well-controlled studies.^[9,10]^ Therefore, the US Food and Drug Administration announced that prescription medicines or over-the-counter products containing codeine or hydrocodone should not be used by patients under 18 years for the treatment of coughs or colds.^[11]^ Another ingredient, guaifenesin, is an expectorant that stimulates the glands to secrete diluted sputum, aggravating the condition of patients with acute gastroenteritis.^[12]^ Additionally, it has not shown any benefit against the common cold in children, when compared to a placebo.^[13,14]^ Thirdly, carbinoxamine maleate, an ingredient not used in Chinese cold medicine, is an ethanolamine-type antihistamine. The use of this ingredient led to the death of 21 2-year-old children in the United States.^[15]^ As a result, the US Food and Drug Administration prohibited the administration of carbinoxamine-containing medicines to children under 2 years of age.^[16]^ Lastly, ephedrine, which effectively treats nasal congestion by vasoconstricting the nasal mucosa, is known to produce severe, adverse cardiovascular and neurological complications. As a consequence, the 2011 guidelines of the French Society of Otorhinolaryngology do not recommend the intake of ephedrine and pseudoephedrine by children under 15 years old. Furthermore, a case report has indicated that long-term use of pseudoephedrine (containing ephedrine analogues) could cause acute renal failure.^[17]^ Which suggesting that the improper use of Taisho Comprehensive Cold Medicine may lead to skin rash and renal impairment. In this case, the child received a lower dose (twice daily) than recommended (3 times daily), the insufficient dosage indicates that the occurrence of the ADR was not related to the medication dosage, but may have been caused by an individual’s immune response to specific drug ingredients.^[18]^

This is the 1st known case of Taisho Comprehensive Cold Medicine-induced drug eruption. However, this case report also has limitation, as the diagnosis was made solely based on clinical symptoms, lack of histopathological biopsy. The cell arrangement, inflammatory cell types, and structural damage conditions reflected by histopathological biopsy can further distinguish different types of rashes and confirm the diagnosis.^[19,20]^ In addition, the cause of rashes can be further determined based on histopathological biopsy, which provide guidance for the formulation of treatment strategies. Thus, it is necessary to conduct a histopathological biopsy.

Although the instructions provided by Taisho Comprehensive Cold Medicine approved its use in children over the age of 1 year, we emphasize that caution is needed when prescribing or administering drugs with similar components to Taisho Comprehensive Cold Medicine to children under 18 years old, since the possible onset of adverse ADRs described in this case report. This report is a powerful public health message about the dangers of using imported, multi-ingredient medications in children, especially when formulations contain substances restricted for pediatric use locally.

Although this case is merely a single incident of adverse reaction caused by a multi-ingredient cold medicine, it has exposed management loopholes in the supervision of individuals’ private purchase of overseas medications. According to the relevant provisions of The Drug Administration Law of the People’s Republic of China, drugs imported without approval, even if they are legally manufactured and therapeutically effective in the country of origin, are still regarded as counterfeit drugs under Chinese law.^[21]^ Therefore, it is illegal for individuals to import such drugs without authorization. These issues not only pose a threat to individual medication safety, but also may present a systemic risk to China’s drug safety system.

## Acknowledgments

The authors would like to thank the patient’s family for giving consent.

## Author contributions

**Conceptualization:** Li Ni.

**Data curation:** Minyan Qian, Li Ni.

**Formal analysis:** Li Ni.

**Funding acquisition:** Li Ni.

**Investigation:** Yingying Tang, Li Ni.

**Methodology:** Li Ni.

**Project administration:** Li Ni.

**Resources:** Li Ni.

**Software:** Li Ni.

**Supervision:** Li Ni.

**Validation:** Na Huang, Mengya Shen, Li Ni.

**Visualization:** Li Ni.

**Writing – original draft:** Li Ni.

**Writing – review & editing:** Yimin Huang, Li Ni.
